# Real‑world evaluation of the efficacy of immune checkpoint inhibitors in the treatment of metastatic breast cancer

**DOI:** 10.3892/ol.2024.14775

**Published:** 2024-10-25

**Authors:** Xiaoyan Qian, Yunxia Tao, Haizhu Chen, Xin Li, Yaqin Wang, Xiaoming Xu, Shuo Li, Haoyu Chen, Shundong Cang, Yang Liu

**Affiliations:** 1 Department of Oncology, Henan Provincial People's Hospital, People's Hospital of Zhengzhou University, People's Hospital of Henan University, Zhengzhou, Henan 450001, P.R. China; 2 Department of Oncology, The Affiliated Hospital of Xuzhou Medical University, Xuzhou, Jiangsu 221000, P.R. China; 3 Guangdong Provincial Key Laboratory of Malignant Tumor Epigenetics and Gene Regulation, Department of Medical Oncology, Breast Tumor Centre, Phase I Clinical Trial Centre, Sun Yat-sen Memorial Hospital, Sun Yat-sen University, Guangzhou, Guangdong 518107, P.R. China; 4 Department of Medical Records, Henan Provincial People's Hospital, People's Hospital of Zhengzhou University, People's Hospital of Henan University, Zhengzhou, Henan 450001, P.R. China; 5 Department of Pharmacy, Henan Provincial People's Hospital, People's Hospital of Zhengzhou University, People's Hospital of Henan University, Zhengzhou, Henan 450001, P.R. China; 6 Department of Medical Records, National Cancer Center/National Clinical Research Center for Cancer/Cancer Hospital and Shenzhen Hospital, Chinese Academy of Medical Sciences and Peking Union Medical College, Shenzhen, Guangdong 518116, P.R. China; 7 Shenzhen MoZhou Tech Co., Ltd., Shenzhen, Guangdong 518057, P.R. China; 8 Department of Medical Oncology, National Cancer Center/National Clinical Research Center for Cancer/Cancer Hospital and Shenzhen Hospital, Chinese Academy of Medical Sciences and Peking Union Medical College, Shenzhen, Guangdong 518116, P.R. China

**Keywords:** breast cancer, immunotherapy, immune checkpoint inhibitor, survival, prognostic factor

## Abstract

The present study aimed to assess the efficacy and safety of immune checkpoint inhibitor (ICI)-based therapy in patients with metastatic breast cancer (MBC). Therefore, eligible patients with histologically confirmed MBC, treated with ICI-based therapy, were enrolled. The primary endpoint was progression-free survival (PFS) and the secondary endpoints included objective response rate (ORR), disease control rate (DCR), overall survival (OS) and safety. A total of 90 patients with MBC, treated with ICI-based therapy, with different treatment lines, were included in the present study. The median age was 50 years (range, 27–76). The predominant tumor subtypes were triple negative (53.3%) and luminal (31.1%) breast cancer. The majority of patients (61.1%) were heavily pretreated (lines of treatment, ≥3). Approximately half of the patients (46.7%) had ≥3 metastatic sites. The overall ORR was 36.7% (33/90 patients), while a DCR of 78.9% (71/90 patients) was also recorded. With a median follow-up of 16.0 months, the median PFS and OS were 4.9 months [95% confidence interval (CI), 3.8–6.1] and 13.9 months (95% CI, 9.5–18.2), respectively. Patients treated with ICIs as first-line therapy exhibited notable improvement, with a median PFS of 11.0 months (95% CI, 6.0–16.0) and a median OS of 24.3 months (95% CI, 11.4–37.2). In addition, the pretreatment blood platelet-to-lymphocyte ratio was an independent risk factor for PFS [hazard ratio (HR)=2.406; 95% CI, 1.325–4.370; P=0.004] and OS (HR=2.376; 95% CI, 1.059–5.328; P=0.036). The most common adverse events were nausea (44.4%), neutropenia (42.0%) and alanine aminotransferase/aspartate aminotransferase elevation (22.2%). Furthermore, three (3.3%) patients developed grade 1/2 immuno-related toxicity and recovered after supportive care. Overall, the present study suggested that the ICI-based therapy exhibited encouraging clinical outcomes with manageable toxicity in patients with MBC in real-world settings, with the most favorable efficacy in first-line treatment.

## Introduction

Breast cancer poses a significant risk to women's health worldwide (1–3). The recent estimates from the International Agency for Research on Cancer demonstrated that breast cancer represents 11.6% of all new cancer cases and 6.9% of all cancer-associated mortalities, with 2.3 million new cases diagnosed and 1.6 million mortalities annually (1). Although significant progress has been made in the treatment of breast cancer, a considerable number of patients experience disease recurrence and metastasis. Consequently, strategies for improving treatment responses and survival outcomes in patients with metastatic breast cancer (MBC) are urgently needed.

The application of immune checkpoint inhibitors (ICIs) is a novel and powerful treatment approach and its efficacy has been verified in several types of solid tumors, such as lung, esophageal and nasopharyngeal cancer and so on (4–6). The available evidence suggests that combination immunotherapy using programmed cell death-1 (PD-1) or programmed cell death-ligand 1 (PD-L1) inhibitors may be a promising treatment option for metastatic triple-negative breast cancer (mTNBC) (7). KEYNOTE 119 trial reported suboptimal efficacy of ICI monotherapy in patients with MBC, thus promoting the investigation of novel therapeutic combinations, including ICIs combined with chemotherapy and/or angiogenic inhibitors (8). In a number of clinical trials, the overall response rate and median overall survival (OS) were improved in patients with mTNBC treated with PD-1/PD-L1 inhibitors in combination with chemotherapy compared with those treated with chemotherapy alone (7,9,10). The IMpassion130 and KEYNOTE-355 clinical trials show notable efficacy of ICI combined with chemotherapy as a first-line therapy for mTNBC (9,10). However, in the the IMpassion131 clinical trial atezolizumab plus paclitaxel failed to demonstrate a significant improvement in progression-free survival (PFS) or OS in mTNBC (11). In the second and later-line settings, ICI combined with angiogenic inhibitors and chemotherapy showed particular efficacy for patients that are heavily pretreated (12). However, clinical evidence for patients with MBC is currently limited. To the best of our knowledge, there are no real-world reports on the value of ICI-based therapy in patients with MBC. Although real-world data lack a well-controlled efficacy design compared with clinical trials, it can provide novel insights into authentic clinical settings and treatment outcomes.

Therefore, in the present study, the characteristics and prognoses of patients with MBC were recorded and the efficacy and safety of ICI-based therapy in distinct treatment lines were evaluated.

## Materials and methods

### Patient eligibility and data collection

In the present study, the data from patients diagnosed with MBC between August 1, 2018 and September 5, 2022, at the Henan Provincial People's Hospital (Zhengzhou, China) and the Affiliated Hospital of Xuzhou Medical University (Xuzhou, China), were retrospectively analyzed. The medical records were collected and assessed from November 2022 to May 2023 in the Henan Provincial People's Hospital and from December 2022 to June 2023 in The Affiliated Hospital of Xuzhou Medical University.

The full indications for ICI treatment in the aforementioned hospitals were as follows: i) Patients with stage II–III TNBC, ICI treatment was used in neoadjuvant setting; ii) patients with TNBC who achieved pathological complete response (pCR) continued to receive ICI treatment in adjuvant setting; iii) patients with TNBC who did not achieve pCR, ICI treatment in adjuvant setting was considered; iv) patients with PD-L1 positive, locally advanced or MBC; v) patients with microsatellite instability-high (MSI-H), locally advanced or MBC; vi) patients with contraindications to chemotherapy and were strongly willing to try ICI treatment; and vii) heavily pretreated patients (lines of treatment, ≥3), with few alternative treatment options and were strongly willing to try ICI treatment.

The inclusion criteria for eligible patients were as follows: i) Patients with histologically confirmed breast cancer according to the World Health Organization classification (13); and ii) patients who were treated with ICI-based therapy in metastatic settings, including ICI plus chemotherapy, ICI plus angiogenic inhibitors and ICI plus chemotherapy and angiogenic inhibitors. The ICIs used included pembrolizumab, atezolizumab, camrelizumab, sintilimab, toripalimab and tislelizumab. The angiogenic inhibitors included anlotinib, apatinib and bevacizumab; and iii) patients with complete clinical and pathological data. The exclusion criteria were as follows: i) Patients who did not receive ICI-based therapy in the metastatic settings; ii) those whose clinical and pathological data were incomplete; and iii) patients without follow-up data. The present study was performed in compliance with the Declaration of Helsinki and was approved by the Institutional Review Board of the Henan Provincial People's Hospital and the Affiliated Hospital of Xuzhou Medical University. Due to the retrospective nature of the study, informed consent from the Institutional Ethics Committee was not required. The clinicopathological data, including age, menstruation status, Eastern Cooperative Oncology Group (ECOG) performance status (PS) score, body mass index (BMI), histological grade, molecular subtype, Ki-67 index, PD-L1 expression, and detailed metastatic information (number of metastatic sites, site of metastatic disease, lines of treatment) were collected. The peripheral blood absolute neutrophil, lymphocyte and platelet (PLT) counts were recorded at the baseline of ICI-based therapy. The neutrophil-to-lymphocyte ratio (NLR) was calculated by dividing the neutrophil count by that of lymphocytes. Consistently, the platelet-to-lymphocyte ratio (PLR) was calculated by dividing the platelet count by that of lymphocytes. Therapy strategies, toxicity, response to therapy, date of relapse, death and last follow-up were also recorded. All data were acquired from the electronic medical records, follow-up visits and by telephone.

### Tumor characteristics

The molecular subtype classification was based on immunohistochemistry. Estrogen receptor (ER) and progesterone receptor (PR) levels were assessed using immunohistochemistry and were considered to be positive if ≥1% of cancer cells were stained. HER2-positive tumors were defined as those with an immunohistochemistry score of ≥3 or positively labeled using fluorescence *in situ* hybridization (FISH) (14). TNBC was defined as ER^−^, PR^−^ and HER2-negative breast cancer. HER2-positive breast cancer was defined as HER2-positive using immunohistochemistry or FISH regardless of the ER and PR status, and included luminal B and non-luminal HER2-positive breast cancer. Luminal breast cancer was defined as ER^+^ or PR^+^ and HER2-negative breast cancer.

### Evaluation of tumor response and adverse events

Tumor response was evaluated based on Response Evaluation Criteria in Solid Tumors (RECIST, version 1.1) (15). The objective response rate (ORR) was defined as the percentage of patients achieving complete response (CR) or partial response (PR). The disease control rate (DCR) was defined as the percentage of patients achieving CR, PR or stable disease (SD). Patients assessed as SD with shrinkage target lesions were documented as SD^−^, while patients categorized as SD with enlarged target lesions were recorded as SD^+^. Progression-free survival (PFS) was defined as the interval between the initial ICI-based therapy and the time of progression or death. Additionally, OS was defined as the interval between the initial ICI-based therapy and the time of death from any cause. Finally, toxicity was assessed according to the Common Terminology Criteria for Adverse Events (CTCAE, version 5.0) (16).

### Statistical analysis

The primary endpoint established was PFS and the secondary endpoints included ORR, DCR, OS and toxicity. Efficacy and safety were evaluated in all enrolled patients who were treated with ≥2 cycles of ICI-based therapy. X-tile software (version 3.6.1, medicine.yale.edu/lab/rimm/research/software/, Yale University) was used to obtain the optimal cut-off values for lymphocyte count, PLT, NLR and PLR. PFS and OS curves were calculated using Kaplan-Meier analysis and were compared using log-rank tests. For univariate and multivariate analyses, a Cox proportional hazards regression model was constructed. The multivariate analysis included the significant characteristics from the univariate analysis. The effects of independent prognostic factors on PFS and OS were validated through C-index using the Python lifelines library (version 3.10, python.org/, Python Software Foundation). P<0.05 (two-sided) was considered to indicate a statistically significant difference. All statistical analyses were performed using SPSS Statistics (version 25.0, IBM Corp.) and R software (http://www.r-project.org; version 3.6.2, RStudio, Inc.).

## Results

### Demographics and clinical characteristics

**Figure 1.**
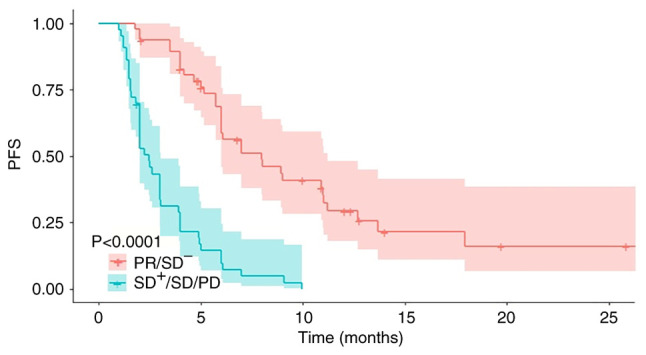
Kaplan-Meier curve for PFS of patients with different tumor responses of immune checkpoint inhibitor-based therapy (PR/SD^−^ vs. SD^+^/SD/PD). PFS, progression-free survival; PR, partial response; SD, stable disease; SD^−^, SD with shrinkage target lesions; SD^+^, SD with enlarged target lesions.

A total of 90 patients treated with ICI-based therapy in the metastatic stage were enrolled and evaluated. Patient demographics and clinical characteristics are shown in Table I. All patients were female. The median age of the patients was 50 years (range, 27–76 years). Among all patients, 80 (88.9%) had an ECOG PS of 0–1, while 39 patients (43.3%) were premenopausal. The predominant tumor subtypes were TNBC (53.3%) and luminal breast cancer (31.1%), while only 7.8% were HER2-positive. In total, 22 (24.4%) patients harbored data on PD-L1 expression, with 13 patients being PD-L1 positive. In addition, the majority of patients (61.1%) were heavily pretreated (lines of treatment, ≥3). Approximately half of patients (46.7%) exhibited ≥3 metastatic sites. The rate of each therapeutic combination was different. More specifically, 44.4% of patients received chemoimmunotherapy, 13.3% received immunotherapy plus angiogenic inhibitors, 28.9% received chemoimmunotherapy plus angiogenic inhibitors, while the remaining 13.3% of patients received a combination of immunotherapy with endocrine or anti-HER2 therapy. In total, 33 patients were confirmed as PR and 38 as SD, while 19 patients were confirmed with progressive disease (PD), yielding an ORR and a DCR of 36.7 and 78.9%, respectively (Table II). No patient was confirmed as CR. The median follow-up time of the total patient cohort was 16.0 months. In the metastatic settings the median lines of treatment was 3, the overall median PFS was 4.9 months (95% CI, 3.8–6.1), whereas the overall median OS was 13.9 months (95% CI, 9.5–18.2). The effect of tumor response on long-term efficacy is shown in Fig. 1. Patients who achieved PR or SD^−^ displayed a median PFS of 6.1 months, which was significantly longer compared with patients classed as SD^+^ or SD or PD [median PFS, 2.1 months; hazard ratio (HR)=5.42; 95% CI, 3.22–9.12; P<0.001].

### Efficacy of distinct lines of treatment

#### Efficacy of the first-line treatment

**Figure 2.**
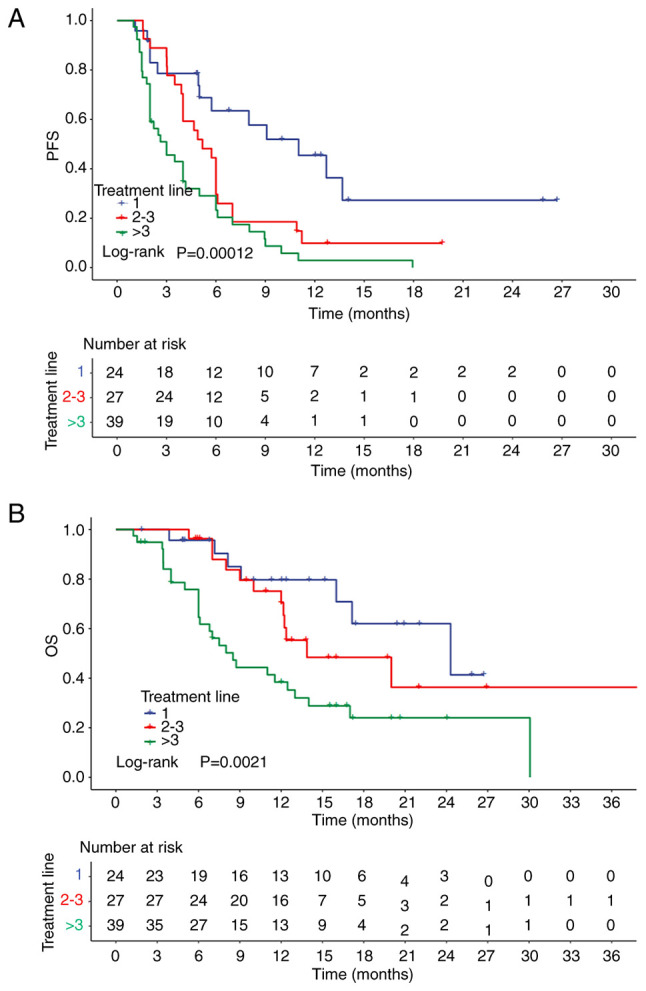
(A) PFS and (B) OS of patients receiving ICI-based therapy in different treatment lines. PFS, progression-free survival; OS, overall survival; ICI, immune checkpoint inhibitor.

A total of 24 patients with breast cancer received ICI-based therapy as first-line therapy. The majority of the patients (23/24) were treated with chemoimmunotherapy, while one patient received immunotherapy plus interleukin-7. The ORR and DCR of each different line of therapy are listed in Table II. In the first-line therapy group, the ICI-based therapy was effective in half of the patients (50.0%; 12/24), as evidenced by the corresponding ORR of 50.0%. In addition, the DCR in the first-line treatment was 91.7% (22/24). The median follow-up time was 15.2 months. The first-line median PFS and OS were 11.0 months (95% CI, 6.0–16.0) and 24.3 months (95% CI, 11.4–37.2), respectively. The PFS and OS of patients treated with distinct lines of ICI-based therapy are shown in Fig. 2.

### Efficacy of the second- and third-line treatment

A total of 27 patients received ICI-based therapy in the second- or third-line setting, including 11 patients in the second-line setting and 16 in the third-line setting. Among them, 10 patients received chemoimmunotherapy, 10 received chemoimmunotherapy plus angiogenic inhibitors and 6 received immunotherapy plus angiogenic inhibitors, while 1 patient was treated with immunotherapy plus endocrine therapy. The overall ORR and DCR in the second- and third-line groups were 55.6 (15/27) and 81.5% (22/27), respectively (Table II). The median follow-up time was 15.4 months, with a median PFS of 5.2 months (95% CI, 3.4–7.0) and a median OS of 13.9 months (95% CI, 4.9–22.8) (Table II).

### Efficacy in later-line treatment

A total of 39 patients were treated with ICI-based therapy in the fourth or later treatment lines. Among them, 16 patients received chemoimmunotherapy plus angiogenic inhibitors, seven received chemoimmunotherapy, seven received immunotherapy plus angiogenic inhibitors, eight received immunotherapy in combination with anti-HER2 agents, while the remaining patients received immunotherapy in combination with endocrine therapy. The overall ORR in these heavily pretreated patients was 38.5% (15/39) and the DCR was 66.7% (26/39) (Table II). Additionally, a median follow-up time of 17.2 months was recorded. The median PFS was 3.0 months (95% CI, 1.5–4.5), while the median OS was 8.5 months (95% CI, 6.1–11.0) (Table II).

### Efficacy of different therapy strategies

A total of 40 patients received immunotherapy combined with chemotherapy. The most commonly used chemotherapy regimen was taxane with or without platinum (75.0%; 30/40; Supplementary Table I). In addition, 57.5% (23/40) of patients were treated with first-line therapy, and 67.5% (27/40) had one or two metastatic sites. The ORR and DCR in the aforementioned patients were 47.5% (19/40) and 82.5% (33/40), respectively. With a median follow-up time of 15.2 months, a median PFS and OS of 7.0 (95% CI, 5.6–8.4) and 24.3 (95% CI, 14.3–34.3) months, respectively, were recorded. Furthermore, a total of 26 patients received chemoimmunotherapy plus angiogenic inhibitors, with a median of four lines of treatment. Among them, 76.9% (20/26) had ≥3 metastatic sites. The ORR and DCR in these heavily pretreated patients were 23.1% (6/26) and 80.8% (21/26), respectively. With a median follow-up time of 16.0 months, a median PFS of 4.0 months (95% CI, 2.9–4.9) and a median OS of 12.0 months (95% CI, 7.9–16.1) were recorded. Additionally, a total of 12 patients were treated with immunotherapy plus angiogenic inhibitors, with a median of four lines of treatment. The most commonly used angiogenic inhibitor was anlotinib (58.3%; 7/12; Table SI). The ORR and DCR were 33.3% (4/12) and 50.0% (6/12), respectively. A median follow-up time of 17.2 months was also recorded, with a median PFS of 2.0 months (95% CI, 0.2–3.8) and a median OS of 12.2 months (95% CI, 11.1–13.3) (Table SII).

### Predictive factors of PFS and OS in ICI-based therapy

The univariate and multivariate analysis results of the factors that were associated with PFS in patients treated with ICI-based therapy are shown in Table III. The univariate analysis demonstrated that BMI, number of metastatic sites, liver metastases, brain metastases, lymphocyte count, PLR, lines of treatment and the choice of systemic treatment were significantly associated with PFS. Due to the overlap between lymphocyte count and PLR, only PLR was included in the multivariate analysis. Finally, liver metastases (HR=2.234; 95% CI, 1.196–4.171; P=0.012), PLR (HR=2.406; 95% CI, 1.325–4.370; P=0.004) and lines of treatment (3 vs. 1; HR=3.531; 95% CI, 1.311–9.512; P=0.013) were identified as independent risk factors in multivariate analysis. The C-index value of the effect of the aforementioned independent prognostic factors on PFS was 0.783 (Table III). Furthermore, the univariate and multivariate analysis results of the factors associated with OS are shown in Table IV. The univariate analysis showed that a higher BMI (>21.5) and lymphocyte count (>1.16), lower PLT (<325) and PLR (≤190), no liver metastases, <3 metastatic sites and <3 treatment lines were notably associated with improved OS. Additionally, the multivariate analysis demonstrated that PLR was a significant covariate for OS (HR=2.376; 95% CI, 1.059–5.328; P=0.036). Finally, the C-index of the effect of the aforementioned independent prognostic factors on OS was 0.723 (Table IV).

### Breast cancer brain metastases

In total, 21 patients had brain metastases (Table I) and 81.0% of them were heavily pretreated (lines of treatment, ≥3). The ORR was 14.3% (3/21) and the DCR was 71.4% (15/21). The median PFS was 3.9 months (95% CI, 2.8–5.2) and the median OS was 11 months (95% CI, 6.2–15.8) (Table SIII).

### Toxicity

The treatment-related adverse events are listed in Table V. Treatment-related mortality was not recorded. The most common adverse events were nausea (44.4%), neutropenia (42.0%) and enhanced alanine aminotransferase/aspartate aminotransferase levels (22.2%). A total of two patients developed immune-related pneumonia (grade 2), while one patient was diagnosed with immune-related myositis (grade 1). All three patients recovered following supportive therapy and continued ICI-based therapy.

## Discussion

Emerging evidence has suggested that ICIs exhibit encouraging antitumor effects on patients with lung cancer, melanoma and other solid carcinomas (17–19). However, breast cancer is commonly classified as a cold immunogenic cancer (20). To the best of our knowledge, the present study was the first multi-center real-world objective report that evaluated the efficacy and safety of ICIs combined with angiogenic inhibitors and/or chemotherapy in China. The results indicated that ICIs could provide positive clinical outcomes with manageable toxicity in patients with MBC in real-world settings. Additionally, the most favorable results were observed in the first-line treatment scenarios.

A previous review evaluated different treatment strategies for metastatic TNBC and summarized that in numerous clinical trials, the ORR and survival were improved in patients treated with ICI treatment combinations compared with those treated with chemotherapy alone (7). The ICI combination may be a promising treatment option for metastatic TNBC. The present study reported the efficacy of ICI treatment combinations in MBC and the clinical outcomes were also improved compared with trials of the aforementioned review where patients were treated with chemotherapy alone, which was consistent with the conclusion of the present review.

The present study assessed the efficacy of ICI-based therapy in patients with MBC among patient groups with various lines of treatment. In the first-line group, the majority of patients (95.8%) underwent immunochemotherapy and reached an ORR of 50.0% and a median PFS of 11.0 months (95% CI, 6.0–16.0), which was consistent with the previous studies (7,9,10). The outcomes were improved compared with later lines of treatment. This may be due to the fact that the immune system could be more effective in patients with primary metastasis compared with heavily pretreated patients (21). Previous preclinical and clinical studies demonstrated that tumor burden is a negative predictor of immunotherapy efficacy, since larger tumors tend to exert a more suppressive microenvironment (22–25). Other clinical studies also verified the efficacy of immunochemotherapy in previously untreated patients with MBC (9,10). Therefore, immunochemotherapy may be a promising alternative in the first-line treatment of MBC. There are limited treatment options for heavily pretreated patients with multiple metastases. Preclinical studies indicate that the angiogenic inhibitor-induced tumor vascular normalization can sensitize programmed cell death PD-1/PD-L1 blockade in several solid tumor models, including breast cancer (26,27). Therefore, immunotherapy combined with angiogenic inhibitors has gained increasing attention as a novel therapeutic strategy.

A phase II study, enrolling 46 patients, reported that combination therapy with camrelizumab, apatinib and eribulin in patients with heavily pretreated advanced TNBC results in an ORR of 37.0%, a DCR of 87.0% and a median PFS of 8.1 months, accompanied by manageable toxicity profile (12). The aforementioned study demonstrated that the novel triplet rationale of ICI, angiogenic inhibitor and chemotherapy warrants further investigation. In the present study, 26 patients received the aforementioned triplet rationale. However, the outcomes of PFS and OS were not as beneficial as that reported in the aforementioned study. This could be due to the difference in the patient population between these two studies. While in the phase II study, all patients were diagnosed with TNBC, in the present study, only 50.0% (13/26) of patients were diagnosed with TNBC. Additionally, in the present study, more patients (43.3%) received ≥3 lines of treatment in the metastatic settings prior enrolment compared with the previous study (37.0%). Overall, the aforementioned findings suggested that the triplet rationale of immunotherapy combined with angiogenic inhibitors and chemotherapy could be a novel and promising strategy for patients with heavily pretreated MBC who underwent multiple lines of unsuccessful systemic therapies. However, the patient population that could benefit most from the aforementioned therapy combinations should be further explored.

Given the synergistic effect of ICIs and angiogenic inhibitors, numerous studies have investigated the efficacy of the combination of ICIs and angiogenic inhibitors. Previous clinical trials showed that the aforementioned chemotherapy-free regimen displayed a particular efficacy, with an ORR of 43.3% for camrelizumab plus apatinib and 29% for pembrolizumab plus lenvatinib (28,29). A phase Ib study, including 34 patients, explored the efficacy of the PD-L1 inhibitor TQB2450 combined with anlotinib. In the aforementioned study, ~80% of patients were treated with a chemotherapy-free regimen as a first- or second-line therapy. The results demonstrated an ORR of 26.5% and a median PFS of 5.6 months (30). Considering the lower toxicity, the chemotherapy-free regimen may be an alternative for patients with poor tolerance.

The present study results showed that the short-term tumor response to immunotherapy could, to a certain extent, predict long-term efficacy. Patients with a shrinkage of target lesions (PR/SD) during immunotherapy exhibited a significantly reduced risk of recurrence compared with others, thus verifying the durable response of immunotherapy. It has been also reported that immunotherapy can prompt a long-term benefit in multiple solid tumors. In a pooled analysis of the CheckMate 017 and 057 trials, 78% of nivolumab-treated patients with non-small-cell lung cancer (NSCLC), who survived for 5 years, showed CR or PR (31). Additionally, the CheckMate-649 trial also demonstrated notably improved outcomes in patients with gastric cancer (32). Therefore, the 3-year OS rate of a subgroup of Chinese patients with PD-L1 combined positive score of 5 in the nivolumab combination with chemotherapy arm was 31%, which was notably increased compared with that obtained in the chemotherapy arm (11%). Numerous studies have also highlighted the unique characteristics of immunotherapy action, namely the ‘tail effect’. Therefore, once the treatment is effective, the long-term survival can be improved, thus providing novel insights into the clinical application of immunotherapy (33,34).

Subgroup analysis demonstrated that high pretreatment blood PLR was associated with poor PFS and OS. Given that peripheral blood testing is simple and easy to perform, PLR could be used as a prognostic factor in patients with MBC treated with ICI-based therapy. In terms of the underlying mechanism, preclinical studies indicated that platelet activation could suppress the functions of natural killer cells and could play a significant role in promoting tumor proliferation, invasion and angiogenic signaling (35,36). Additionally, lymphocytes can inhibit tumor cell growth and improve the prognosis of patients with malignant tumors via secreting IFN-g and TNF (37–41). Emerging evidence has also suggested that PLR is an informative marker reflecting the changes in platelet and lymphocyte counts, while it is associated with the degree of inflammation (42). Therefore, PLR may potentially exhibit a negative effect on the immune system of the host (43). Other studies have also suggested that a high PLR may be a negative immunotherapy prognostic factor in several types of solid tumors, such as NSCLC and gastric cancer (44,45). A previous study by Onagi *et al* (43) demonstrated that in patients with TNBC, a high PLR predominantly includes more CD3^+^CD4^+^FOXP3^+^ T-cells (regulatory T-cells), thus suggesting that local tumor immunity can be suppressed in these patients, eventually leading to poor immunotherapy outcomes.

Of note, two real-world studies also explored the efficacy of ICIs in MBC. The ANASTASE study (46), which retrospectively enrolled 52 patients with PD-L1-positive metastatic TNBC treated with first-line atezolizumab plus nab-paclitaxel, achieved an ORR of 42.3% and a median PFS of 6.3 months; while in the present study, 24 patients of first-line therapy, regardless of PD-L1 level, reached an ORR of 50% and a median PFS of 11 months. In contrast to the ANASTASE study, the present study not only included patients who received Nab-paclitaxel + immunotherapy (12 patients), but also stronger combinations, such as platinum-based chemotherapy or anthracyclines plus taxane, which might be the reason of longer PFS. Another real-world study (47) in China included 81 patients with metastatic TNBC, of which 9.9% patients received ICI monotherapy and 90.1% patients received combined treatment. The ORR was 32.1% and the DCR was 64.2%. The median PFS was 4.2 months and the median OS was 11.0 months. The combined drugs were not described specifically. The present study classified therapy strategies and reported their outcomes respectively, which shed light on the exploration of different combination of ICIs. A systematic review (48) concluded that up to 45% of patients with TNBC will develop breast cancer brain metastases (BCBM) but only 3.3% of patients were included in the clinical trials, and only two clinical trials have reported the BCBM-specific outcomes. Evaluation of the efficacy of ICIs in patients with BCBM is greatly needed. The present study reported the survival outcomes of patients with BCBM and provided more evidence to patients with BCBM.

However, the present study has a number of limitations. Due to its retrospective nature, potential biases should be taken into consideration, including no controlled-arm, patient selection and over-enthusiasm, which describes a potential bias introduced when clinical physicians pay more attention to positive outcomes or improvements in patients receiving treatment and may inadvertently overlook or underreport adverse events or negative outcomes. In addition, clinical physicians potentially might pay more attention to positive outcomes in patients receiving ICIs, inadvertently overlooking or underreporting adverse events or negative outcomes.

Breast cancer was previously not considered a particularly immunogenic tumor due to relatively lower levels of tumor infiltrating lymphocytes, tumor mutational burden and PD-L1 expression (49–51). Nevertheless, there is increasing evidence in recent years to suggest the presence of variable immunogenic activity in different breast cancer subtypes, with TNBC likely exhibiting the strongest immunogenicity (52,53). However, as the ICIs approved by the Food and Drug Administration (FDA) for the treatment of breast cancer, namely atezolizumab and pembrolizumab, are expensive and not covered by medical insurance, immunotherapy for the treatment of breast cancer has not been prioritized clinically. As a result, the application of immunotherapy is rare in the real world. Therefore, the present study included a heterogeneous patient population and certain confounding factors were unavoidable, such as patients using a variety of ICIs, including pembrolizumab, atezolizumab, camrelizumab, sintilimab, toripalimab and tislelizumab. By contrast, this allowed the analysis of the comprehensive subgroups of different combinations, such as ICI combined with chemotherapy, ICI with angiogenic inhibitors and ICI with chemotherapy and angiogenic inhibitors.

However, not all patients have finished PD-L1 detection, for a number of reasons including limited biopsy tissue, low quality tissue samples as samples that must comprise sufficient amounts of tumor cells, lymphocytes and phagocytes and as certain patients started immunotherapy before PD-L1 was recognized as a clinically relevant biomarker for ICIs in November 2020, when the US FDA accelerated the approval of pembrolizumab in combination with chemotherapy based on the positive outcomes of KEYNOTE 355. Several patients did not assess PD-L1 but still received ICIs. The reasons were as follows: i) Several patients harbored high mutational burden or microsatellite instability, which might make ICIs a reasonable treatment option; ii) there might be patient-specific factors that made ICIs a preferred treatment option, such as comorbidities that make other treatments less suitable; and iii) in certain cases, ICIs might be used off-label based on the judgment of the clinical physician, considering the potential benefits and risks, especially for patients whose cancer has progressed after other treatments.

Machine learning was not applied in the present study, as machine learning often cannot achieve sufficiently reliable results when the sample data is not large enough and also involves a large number of parameters. For a model with multiple factors, it often requires thousands or even tens of thousands of data points to generate a model with some effectiveness. In cases where the data volume is small, the data dimensions are high, statistical methods tend to be more efficient and effective than machine learning (54,55).

Despite the aforementioned limitations, the findings of the present study could still be considered meaningful as it provided real-world outcomes of ICI-based therapy in MBC and offered insight on the role and application of ICIs in MBC. However, to enforce global policy changes, large-scale phase III clinical trials are needed. Multi-centered, randomized controlled prospective trials could be conducted to confirm the efficacy and safety of ICIs in the first-line treatment of MBC, particularly for ICIs developed in China, such as camrelizumab, sintilimab, toripalimab and tislelizumab. Further investigation on the role of baseline PLR as a prognostic biomarker for ICI treatment and additional biomarkers or factors that could predict response to ICI treatment are also warranted. In addition, investigations of combination therapies that could enhance the effectiveness of ICIs should also be considered in the future.

In conclusion, the present study demonstrated that ICI-based therapy could achieve promising clinical outcomes with manageable toxicity for patients with MBC in real-world settings. The most appropriate time for ICIs was the first-line treatment. Furthermore, baseline PLR could serve as an independent prognostic factor for ICI treatment. In addition, it was demonstrated that once the ICI treatment was effective, long-term survival could be improved. However, although the present study had also a number of limitations, the preliminary findings of this real-world cohort warrant further prospective research and clinical practice of treating MBC in future.

## Supplementary Material

Supporting Data

## Figures and Tables

**Table I.** Baseline characteristics of patients.

Baseline characteristics	Patients
Median age, years (range)	50 (27–76)
Age group, n (%)	
≤47 years	37 (41.1)
>47 years	53 (58.9)
ECOG PS, n (%)	
0-1	80 (88.9)
2	10 (11.1)
Body mass index, n (%)	
≤21.5	26 (28.9)
>21.5	64 (71.1)
Menopausal state, n (%)	
Postmenopausal	51 (56.7)
Premenopausal	39 (43.3)
Pathology, n (%)	
Invasive ductal carcinoma	85 (94.4)
Metaplastic carcinoma	4 (4.4)
Neuroendocrine carcinoma	1 (1.1)
Histology type, n (%)	
I	1 (1.1)
II	22 (24.4)
III	36 (40.0)
Unknown	31 (34.4)
Molecular subtypes, n (%)	
Triple-negative breast cancer	48 (53.3)
Luminal^a^	28 (31.1)
HER2-positive^b^	7 (7.8)
Unknown	7 (7.8)
Ki-67 index, n (%)	
≤50%	32 (35.6)
>50%	51 (56.7)
Unknown	7 (7.8)
No. of metastatic sites, n (%)	
1-2	48 (53.3)
≥3	42 (46.7)
Site of metastatic disease, n (%)	
Brain	21 (23.3)
Lung	51 (56.7)
Liver	33 (36.7)
Bone	40 (44.4)
Lymphocyte count, n (%)	
≤1.16	47 (52.2)
>1.16	42 (46.7)
Platelet count, n (%)	
<325	72 (80.0)
≥325	17 (18.9)
Neutrophil to lymphocyte ratio, n (%)	
≤3.16	55 (61.1)
>3.16	34 (37.8)
Platelet-to-lymphocyte ratio, n (%)	
≤171	34 (37.8)
>171	55 (61.1)
Lines of treatment, n (%)	
1	24 (26.7)
2-3	27 (30.0)
>3	39 (43.3)
Choice of systemic treatment, n (%)	
ICI plus chemotherapy	40 (44.4)
ICI plus angiogenic inhibitors	12 (13.3)
ICI plus chemotherapy and angiogenic inhibitors	26 (28.9)
ICI plus others	12 (13.3)
PD-L1 expression, n (%)	
<1%	9 (10.0)
≥1%	13 (14.4)
Unknown	68 (75.6)

a Luminal breast cancer included luminal A and B HER2-negative tumors;

b HER2-positive included luminal B and non-luminal HER2-positive tumors. ECOG PS, Eastern Cooperative Oncology Group performance status; ICI, immune checkpoint inhibitor; PD-L1, programmed cell death-ligand 1.

**Table II.** Efficacy of distinct lines of treatment.

Treatment line	No. of patients	Median follow-up time (months)	PFS (median, 95% CI) (months)	OS (median, 95% CI) (months)	ORR, %	DCR, %
Total patient cohort	90	16.0	4.9 (3.8–6.1)	13.9 (9.5–18.2)	36.7 (33/90)	78.9 (71/90)
First-line	24	15.2	11.0 (6.0–16.0)	24.3 (11.4–37.2)	50.0 (12/24)	91.7 (22/24)
Second and third-line	27	15.4	5.2 (3.4–7.0)	13.9 (4.9–22.8)	55.6 (15/27)	81.5 (22/27)
Fourth or later-line	39	17.2	3.0 (1.5–4.5	8.5 (6.1–11.0)	38.5 (15/39)	66.7 (26/39)

PFS, progression-free survival; OS, overall survival; ORR, objective response rate; DCR, disease control rate.

**Table III.** Univariable and multivariate analysis of predictive factors for PFS.

	Univariate analysis		Multivariate analysis		
			
Baseline characteristics	HR (95% CI)	P-value	HR (95% CI)	P-value	C-index
Age group, years					0.783
≤47	Reference				
>47	0.945 (0.574–1.505)	0.813			
ECOG PS					
0-1	Reference				
2	1.228 (0.758–1.989)	0.404			
Body mass index					
≤21.5	Reference		Reference		
>21.5	0.490 (0.292–0.823)	0.007	0.844 (0.424–1.677)	0.628	
Menopausal state					
Postmenopausal	Reference				
Premenopausal	0.861 (0.541–1.371)	0.529			
Pathology					
Invasive ductal carcinoma	Reference				
Non-invasive ductal carcinoma	2.157 (0.862–5.398)	0.100			
Histology type					
I–II	Reference				
III	1.205 (0.658–2.205)	0.546			
Molecular subtypes					
Non-TNBC	Reference				
TNBC	0.807 (0.765–2.009)	0.384			
Ki-67 index					
≤50%	Reference				
>50%	1.525 (0.926–2.511)	0.097			
No. of metastatic sites					
1-2	Reference		Reference		
≥3	2.050 (1.261–3.333)	0.004	0.945 (0.43–2.075)	0.888	
Lung metastasis					
No	Reference				
Yes	1.506 (0.935–2.424)	0.092			
Liver metastasis					
No	Reference		Reference		
Yes	2.373 (1.461–3.854)	<0.001	2.234 (1.196–4.171)	0.012	
Brain metastasis					
No	Reference		Reference		
Yes	1.791 (1.054–3.041)	0.031	1.403 (0.662–2.976)	0.377	
Lymphocyte count					
≤1.16	Reference				
>1.16	0.425 (0.264–0.685)	<0.001			
Platelet count					
<325	Reference				
≥325	1.310 (0.743–2.311)	0.351			
Neutrophil-to-lymphocyte ratio					
≤3.16	Reference				
>3.16	1.566 (0.984–2.494)	0.059			
Platelet-to-lymphocyte ratio					
≤190	Reference		Reference		
>190	2.309 (1.426–3.739)	0.001	2.406 (1.325–4.370)	0.004	
No. of lines of treatment					
1	Reference		Reference		
2-3	2.163 (1.092–4.283)	0.027	1.855 (0.799–4.307)	0.151	
>3	3.646 (1.907–6.972)	<0.001	3.531 (1.311–9.512)	0.013	
Choice of systemic treatment					
ICI plus chemotherapy	Reference		Reference		
ICI plus angiogenic inhibitors	3.386 (1.645–6.969)	0.001	2.376 (0.977–5.779)	0.056	
ICI plus chemotherapy and angiogenic inhibitors	2.482 (1.387–4.441)	0.002	0.821 (0.361–1.866)	0.638	
PD-L1 expression, %					
<1	Reference				
≥1	0.867 (0.331–2.268)	0.771			

PFS, progression-free survival; HR, hazard ratio; CI, confidence interval; ECOG PS, Eastern Cooperative Oncology Group performance status; TNBC, triple-negative breast cancer; ICI, immune checkpoint inhibitor; PD-L1, programmed cell death-ligand 1.

**Table IV.** Uni- and multivariate analysis of predictive factors for OS.

	Univariate analysis		Multivariate analysis		
			
Baseline characteristics	HR (95% CI)	P-value	HR (95%CI)	P-value	C-index
Age group, years					0.723
≤47	Reference				
>47	0.605 (0.338–1.082)	0.090			
ECOG PS					
0-1	Reference				
2	1.271 (0.693–2.333)	0.439			
Body mass index					
≤21.5	Reference		Reference		
>21.5	0.407 (0.225–0.738)	0.003	0.726 (0.334–1.579)	0.419	
Menopausal state					
Postmenopausal	Reference				
Premenopausal	0.868 (0.478–1.578)	0.643			
Pathology					
Invasive ductal carcinoma	Reference				
Non-invasive ductal carcinoma	1.508 (0.466–4.878)	0.492			
Histology type					
I–II	Reference				
III	0.667 (0.322–1.380)	0.275			
Molecular subtypes					
Non-TNBC	Reference				
TNBC	0.886 (0.483–1.626)	0.699			
Ki-67 index					
≤50%	Reference				
>50%	1.719 (0.909–3.254)	0.096			
No. of metastatic sites					
1-2	Reference		Reference		
≥3	2.267 (1.248–4.119)	0.007	1.092 (0.504–2.363)	0.823	
Lung metastasis					
No	Reference				
Yes	1.576 (0.864–2.872)	0.138			
Liver metastasis					
No	Reference		Reference		
Yes	2.342 (1.298–4.223)	0.005	2.163 (0.976–4.797)	0.058	
Brain metastasis					
No	Reference				
Yes	1.601 (0.833–3.078)	0.158			
Lymphocyte count					
≤1.16	Reference				
>1.16	0.447 (0.244–0.817)	0.009			
Platelet count					
<325	Reference				
≥325	1.978 (1.016–3.853)	0.045			
Neutrophil-to-lymphocyte ratio					
≤3.16	Reference				
>3.16	1.553 (0.867–2.782)	0.139			
Platelet-to-lymphocyte ratio					
≤190	Reference		Reference		
>190	2.203 (1.174–4.134)	0.014	2.376 (1.059–5.328)	0.036	
No. of lines of treatment					
1	Reference		Reference		
2-3	1.558 (0.610–3.974)	0.354	1.784 (0.559–5.692)	0.328	
>3	3.480(1.506–8.043)	0.040	2.784 (0.775–9.992)	0.116	
Choice of systemic treatment					
ICI plus chemotherapy	Reference		Reference		
ICI plus angiogenic inhibitors	2.942 (1.285–6.734)	0.011	1.497 (0.504–4.453)	0.468	
ICI plus chemotherapy and angiogenic inhibitors	1.807 (0.865–3.777)	0.116	0.566 (0.191–1.676)	0.304	
PD-L1 expression, %					
<1	Reference				
≥1	1.296 (0.322–5.220)	0.715			

OS, overall survival; HR, hazard ratio; CI, confidence interval; ECOG PS, Eastern Cooperative Oncology Group performance status; TNBC, triple-negative breast cancer; ICI, immune checkpoint inhibitor; PD-L1, programmed cell death-ligand 1.

**Table V.** Treatment-related toxicity events reported in the patient cohort.

Toxicity event	All grades, n (%)	Grade 3 or 4, n (%)
Nausea	40 (44.4)	0 (0)
Peripheral neuropathy	7 (7.8)	0 (0)
Hypothyroidism	2 (2.2)	0 (0)
Hypertension	3 (3.3)	2 (2.2)
Neutropenia	38 (42)	18 (20)
Thrombocytopenia	3 (3.3)	1 (1.1)
ALT/AST elevation	20 (22.2)	3(3.3)
Pneumonia	2 (2.2)	0 (0)
Myositis	1 (1.1)	0 (0)

ALT, alanine aminotransferase; AST, aspartate aminotransferase.

## Data Availability

The data generated in the present study may be requested from the corresponding author.
