# Supplementary material for: Real‑world evaluation of the efficacy of immune checkpoint inhibitors in the treatment of metastatic breast cancer
Source: Oncol Lett. 2024 Oct 25;29(1):29. doi: 10.3892/ol.2024.14775 (PMC11542155; doi:10.3892/ol.2024.14775)
Supplement: Supporting Data [file Supplementary_Data.pdf]

Table SI. Systemic treatment of patients.

Systemic treatment	No. of patients (%)
ICI plus chemotherapy	40 (44.4)
ICI plus taxane ± platinum	30 (33.3)
ICI plus gemcitabine ± platinum	3 (3.3)
ICI plus vinorelbine ± platinum	3 (3.3)
ICI plus other chemotherapy	4 (4.4)
ICI plus angiogenic inhibitors	12 (13.3)
ICI plus anlotinib	7 (7.8)
ICI plus apatinib	3 (3.3)
ICI plus bevacizumab	1 (1.1)
ICI plus endostar	1 (1.1)
ICI plus chemotherapy and angiogenic inhibitors	26 (28.9)
ICI plus taxane and anlotinib/apatinib	7 (7.8)
ICI plus gemcitabine and anlotinib/apatinib	2 (2.2)
ICI plus vinorelbine and anlotinib/apatinib/ Bevacizumab	4 (4.4)
ICI plus eribulin and apatinib/bevacizumab	2 (2.2)
ICI plus capecitabine and anlotinib/bevacizumab	2 (2.2)
ICI plus other chemotherapy and angiogenic Inhibitors	9 (10.0)
ICI plus others	12 (13.3)
ICI plus trastuzumab	7 (7.8)
ICI plus antibody drug conjugate	1 (1.1)
ICI plus endocrine or IL-7	4 (4.4)

ICI, immune checkpoint inhibitor; IL, interleukin-7.

Table SII. Efficacy of different systemic treatment strategies.

Systemic treatment	No. of patients	median follow-up time (months)	PFS (median, 95%CI) (months)	OS (median, 95%CI) (months)	ORR, %	DCR, %
ICI plus chemotherapy	40	15.2	7.0 (5.6-8.4)	24.3 (14.3-34.3)	47.5 (19/40)	82.5 (33/40)
ICI plus angiogenic inhibitors	12	17.2	2.0 (0.2-3.8)	12.2 (11.1-13.3)	33.3 (4/12)	50.0 (6/12)
ICI plus chemotherapy and angiogenic inhibitors	26	16.0	4.0 (2.9-4.9)	12.0 (7.9-16.1)	23.1 (6/26)	80.8 (21/26)

PFS, progression-free survival; CI, confidence interval; OS, overall survival; ORR, objective response rate; DCR, disease control rate; ICI, immune checkpoint inhibitor.

Table SIII. Efficacy of brain metastases patients (n=21).

PFS (median, 95%CI) (months)	OS (median, 95%CI) (months)	ORR, %	DCR, %
3.9 (2.8-5.2)	11 (6.2-15.8)	14.3 (3/21)	71.4 (15/21)

PFS, progression-free survival; CI, confidence interval; OS, overall survival; ORR, objective response rate; DCR, disease control rate.
